# Predictive value of tip-apex distance and calcar-referenced tip-apex distance for cut-out in 398 femoral intertrochanteric fractures treated in a private practice with dynamic intramedullary nailing

**DOI:** 10.3389/fsurg.2024.1438858

**Published:** 2024-08-14

**Authors:** Amariel E. Barra, Carlos Barrios

**Affiliations:** ^1^ School of Doctorate, Valencia Catholic University, Valencia, Spain; ^2^ Institute for Research on Musculoskeletal Disorders, Valencia Catholic University, Valencia, Spain

**Keywords:** intertrochanteric fractures, dynamic intramedullary nailing, cut-out, tip-apex distance, fracture reduction

## Abstract

**Introduction:**

Cut-out, a biomechanical complication, is one of the most common causes of internal fixation failure of trochanteric hip fractures. The tip-apex distance (TAD) and the calcar-referenced tip-apex distance (CalTAD) have been suggested as the radiographic parameters that most predict the risk of cut-out. The purpose of this study was to check whether these two factors could predict implant cut-out in a series 398 of intertrochanteric hip fractures, treated by dynamic intramedullary nailing with the Trigen Intertan short nail.

**Methods:**

We reviewed 398 consecutive intertrochanteric fractures included in a prospective study and treated in a single private hospital by the same surgeon. The radiographic parameters were obtained from anteroposterior (AP) and axial hip plain radiographs before surgery, immediately postoperatively, and every 3 weeks after surgery until 3 months postoperatively, and every month until the 6-month follow-up. The concept of medial cortex support (MCS) was also analyzed as a criterion for evaluating the quality of fracture reduction.

**Results:**

The overall cut-out rate was 2.3% (9/398). The significant parameters in the univariate analysis were AO fracture type, quality of fracture reduction (*p* = 0.02), TAD (*p* < 0.001), CalTAD (*p* = 0.001), and quality of reduction. No statistically significant relationships were observed between the occurrence of cut-out and sex, age, fracture side, and American Society of Anesthesiologists type. Varus collapse and cut-out were only found in cases of negative MCS (22.2% and 77.8%, respectively). Multivariate analysis showed that only TAD showed an independent significant relationship to cut-out (*p* < 0.001). In this study, CalTAD has no predictive value in the multivariable analysis.

**Conclusions:**

Our findings differed from those in previous reported studies suggesting that CalTAD is the best predictor of cut-out. According to our data, careful optimal reduction ensuring stable fixation with TAD >25 mm reduced the occurrence of cut-out after dynamic intramedullary nailing of intertrochanteric fractures.

## Introduction

Fractures of the proximal femur are always a topic of debate due to their high frequency and socio-sanitary impact, from both medical and socioeconomic perspectives. The incidence of hip fractures in the population aged over 70 years progressively increases every year, being one of the most common causes of morbidity and mortality in the geriatric population (1). Epidemiologically, an incidence of 4.5 million fractures is expected by 2050 (2), reaching a prevalence of approximately 50% of all hip fractures (3). In their treatment, intramedullary nailing has been gaining ground over other systems. For example, in the USA, its usage has progressed from 3% in 1999 to 67% in 2006 (3). However, despite the evolution of implants over time, the mortality rate of the most unstable fractures during the first year of surgery is in the range of 11%–27% (4). Among the most frequent causes of mortality are the so-called biomechanical complications, of which “cut-out” is the most relevant (5, 6).

Cut-out is defined as varus collapse of the fracture at the cervicocephalic angle, leading to extrusion of the cephalic screw outside the femoral head (3). The frequency of this complication varies among studies, between 3.2% and 20.5% (1, 7). However, this complication tends to occur more frequently in older patients with more comorbidities. It is in these patients in particular that surgery for the complication increases the risk of further complications, leading to greater rehabilitation and hospital stay. Therefore, early detection of a cut-out complication would lead to more effective and less injurious treatment than that of an already established complication (8).

Different authors have established a significant relationship between different variables and cut-out, although there is little clear evidence in some of them, such as the quality of the reduction achieved in the operating room (9–11). There is a broad consensus that a reduction of the medial cortex is necessary to avoid cut-out (12–14). If the reduction of the medial cortex is poor, the implant will likely fail. There seem to be new studies that reduce the types of reduction to three, namely anatomical reduction, positive medial cortical support (PMCS), and negative medial cortical support (NMCS), according to the contact between the cervicocephalic fragment and the femoral diaphyseal fragment (12). As anatomical reduction is desired and positive medial cortical support has more biomechanical benefits than reduction with negative medial cortical support, the first two reductions are considered good (13). The quality of reduction according to medial cortical support variations has never been related to cut-out occurrence after hip intertrochanteric fractures treated with dynamic intramedullary nailing.

With this background knowledge, the objective of this study was to assess the relationship between the quality of reduction assessed by the medial cortical support and the occurrence of cut-out. Furthermore, two classic radiographic predictive factors that define the placement of the cephalic screw were also analyzed, namely tip-apex distance (TAD) and calcar-apex distance (CalTAD).

## Materials and methods

### Study design

**Figure 1:**
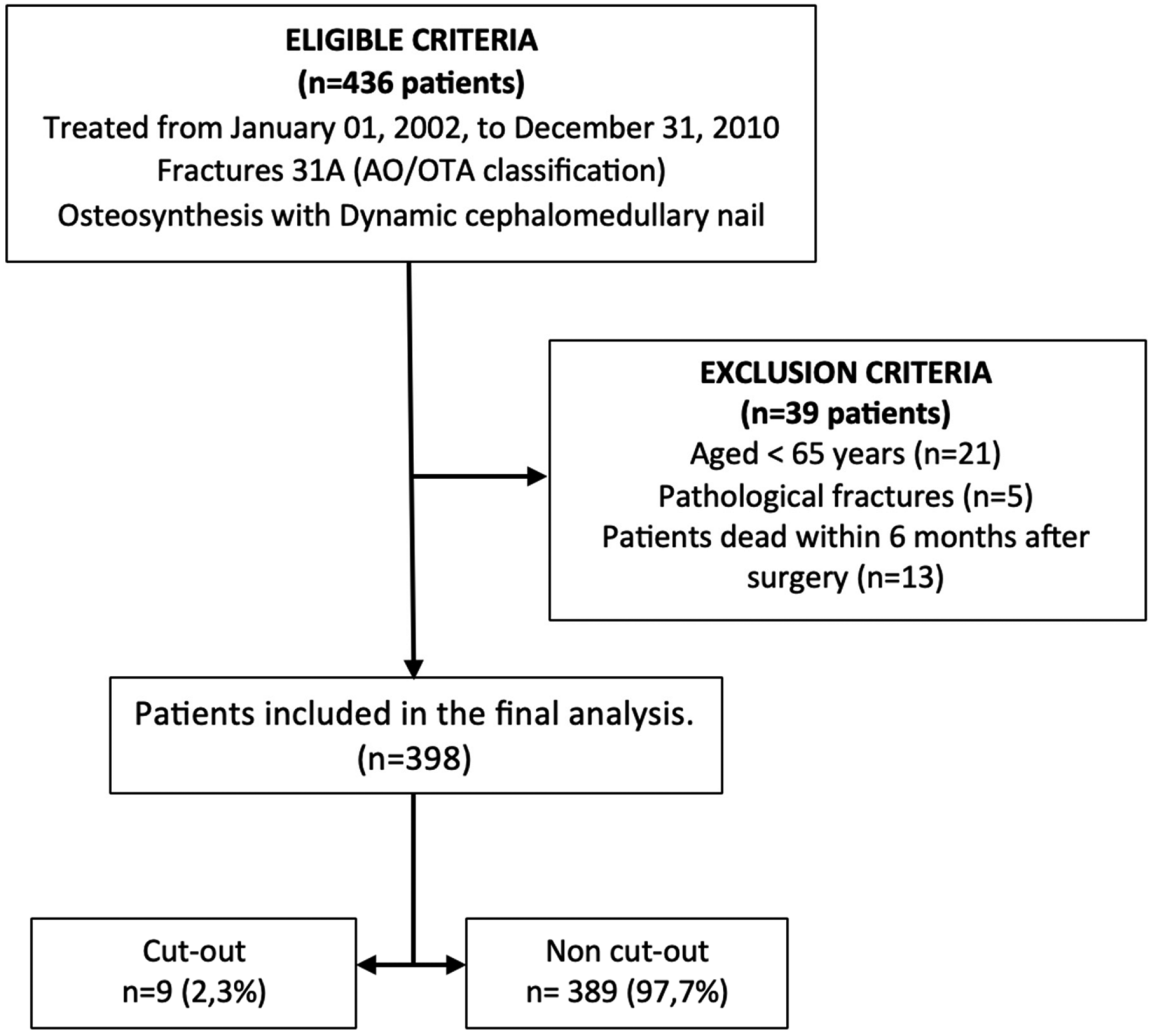
Flow chart describing study inclusion and exclusion criteria.

This was a retrospective analysis of a prospective study on consecutive patients with intertrochanteric femur fracture treated with closed reduction and internal fixation with dynamic intramedullary nailing, admitted to a private hospital (Vithas Valencia Hospital, Valencia, Spain) between January 2002 and December 2010. Only fractures classified as 31A according to the AO-Müller/Orthopaedic Trauma Association (AO/OTA) classification were included (15). Consecutive patients undergoing surgery with the Trigen Intertan® short nail (Intertrochanteric Antegrade Nail; Smith & Nephew, UK) were identified retrospectively from the hospital discharge database (Figure 1).

The exclusion criteria were as follows: patients aged under 65 years; proximal femoral fracture involving femur diaphysis or subtrochanteric fractures; pathological fractures induced by tumors or metastatic lesions; poor-quality radiographs; and patients without a minimum follow-up period of 6 months.

All surgeries were performed on an orthopedic table, preceded by closed reduction of the fracture by the same surgeon. In all cases, distal locking was performed with a single screw. All patients followed a postoperative rehabilitation program consisting primarily of initiating partial weight-bearing standing from postoperative day 1 or as soon as the patient's condition allowed.

### Variables

For each patient, the following data were documented: sex, American Society of Anesthesiologists (ASA) classification, duration of operation, length of hospital stay, type of anesthesia, mortality rate, incidence of cut-outs or other complications, and postoperative weight-bearing.

The radiographic parameters were obtained from anteroposterior (AP) and axial hip plain radiographs before surgery, immediately postoperatively, and every 3 weeks after surgery until 3 months postoperatively, and every month until the 6-month follow-up. The last radiograph check-up was carried out 1 year after surgery. Fractures were distributed according to the AO/OTA classification as types A1, A2, and A3 with the corresponding subtypes (15). In the postoperative radiographs, the following measurements were taken:

• TAD: this is the sum of the distances from the distal end of the cephalic screw to the apex of the femoral head in anteroposterior and lateral projections (16)
• CalTAD: this is the sum of the distance from the distal end of the cephalic screw to the apex of the head in the lateral projection plus the distance from the distal end of the cephalic screw to a line tangential to the medial cortex of the femoral neck (17)

Assessment of intraoperative reduction as satisfactory or unsatisfactory was based on displacement and angulation criteria. Correct displacement is accepted up to 4 mm, and correct angulation is considered a slightly valgus cervicodiaphyseal angle (130°–150°) on the anteroposterior radiograph and less than 20° of varus angulation on the lateral radiograph. Therefore, reduction quality is considered satisfactory if both criteria are met and unsatisfactory if either of them does not match.

The concept of medial cortex support (MCS) was also analyzed as a criterion for evaluating the quality of fracture reduction (12). A positive medial cortex support (PMCS) was defined as the proximal femoral head–neck fragment being displaced superomedially by one cortex thickness from the medial cortex of the femoral shaft. A neutral position (NP) was characterized by anatomical contact between the medial cortex of the head–neck fragment and the shaft fragment. Conversely, a negative medial Cortical support (NMCS) was identified when the head–neck fragment was displaced laterally to the upper medial edge of the shaft fragment, resulting in the loss of medial cortex support from the femoral shaft. Fracture collapse after stabilization was calculated according to Doppelt's method (18).

The collected data were analyzed to find a possible relationship between these radiographic parameters and the occurrence of the dreaded cut-out. The independent variables were divided into non-modifiable (not related to surgery) and modifiable (related to surgery) causes of mechanical failure. Non-surgery-related factors included age, sex, fracture side, ASA anesthetic risk, and fracture type. Surgery-related factors included length of the lag-screw, quality of reduction, TAD, and CalTAD. To assess the relationships of each variable studied with cut-out, we divided the study population into two subgroups: those who did not experience the complication (Group A) and those who did (Group B).

### Ethical considerations

The study was approved by the Research Ethics Committee of Vithas Valencia 9 October Hospital. Data collection and analysis were performed in compliance with the Declaration of Helsinki. The study was designed and described according to STROBE guidelines for reporting observational studies (19).

### Statistical analysis

The Shapiro–Wilk test assessed the normality of distribution for continuous variables. Symmetrical distributions were represented as the mean and standard deviation (SD), while non-normal distributions were expressed as the median and interquartile range (IQR). Categorical data were presented as absolute numbers and percentages. Statistical comparisons of categorical variables employed Pearson's χ^2^ test or Fisher's exact test, depending on minimal expected counts in each cross-tab. Continuous variable comparisons used the one-factor ANOVA test for normally distributed variables and the Mann–Whitney *U*-test for non-normal distributions. Univariate analysis estimated receiver operating characteristic (ROC) curves for TAD and CalTAD to measure testing accuracy. The thresholds for TAD and CalTAD were determined as optimal cut-offs maximizing the distance from the identity line in the ROC curve, based on Youden's J statistic. Two multivariate linear regression models identified factors associated with cut-out presence, one using standard TAD and CalTAD thresholds and the other using thresholds determined in our Youden's analysis. A *p*-value <0.05 indicated statistical significance. All analyses were performed using SPSS version 29.0 (IBM Corp., Armonk, New York, USA).

## Results

A total of 436 patients were initially identified for potential inclusion in the study. However, after screening, only 398 patients fulfilled the inclusion criteria and were deemed eligible for participation. The primary reasons for exclusion included patients aged under 65 years, a follow-up period of less than 6 months after surgery mainly because of death, and instances of pathologic fractures (Figure 1).

Among the 398 patients included in this study, the female-to-male ratio was 4:1 with a mean age of 78.6 years. Most patients were classified as ASA 2 (44.5%) and ASA 3 (44.3%). The most frequent fracture type was A2.2 (45.8%), followed by type A2.3 (43%).

The mean surgical delay was 3.6 days (SD = 2.3). Surgery was performed on the same day of admission for 40 (10.1%) patients. Most patients (79.9%) underwent surgery within the first 5 days after injury. The mean operative time was 35 min (SD = 8.9; range 15–90 min). All patients received subarachnoid regional anesthesia and sedation. The mean length of hospitalization was 13.1 days (SD = 5.3), with stays in the range of 5–46 days. Out of the 398 patients, 13 (3.2%) died in the first 6 months of follow-up.

Among the 398 patients who were reviewed, 30 had a varus displacement of the femoral head and neck without clinical repercussions. Lag-screw cut-out was observed in 9 (2.3%) cases (Table 1), with a female-to-male ratio of 8.1 and a mean age of 71.6 years. All cases of cut-out were ASA 2 (four cases) and ASA 3 (five cases). The fracture pattern most associated with cut-out in this series is shared by fracture type A2.2 (four cases) and type A2.3 (four cases). No statistically significant relationships were observed between the occurrence of cut-out and sex, age, fracture side, and ASA type.

**Table 1:** Clinical and radiological characteristics of cases with cut-out.

Cut-out case	Age	Gender	Side	ASA grade	AO fracture type	Quality of reduction	TAD (mm)	CalTAD (mm)
1	73	F	Right	3	2.2	Non-satisfactory	39.1	41.34
2	75	F	Right	2	2.2	Satisfactory	29.5	30.74
3	78	F	Left	2	2.3	Non-Satisfactory	35.1	37.1
4	79	F	Right	3	2.2	Satisfactory	28.5	29.68
5	79	M	Right	3	3.2	Non-Satisfactory	44.2	46.64
6	83	F	Right	2	2.3	Satisfactory	25.8	27.35
7	83	F	Left	3	2.3	Non-Satisfactory	32.4	34.34
8	85	F	Left	2	2.3	Satisfactory	25.5	27.03
9	93	F	Right	3	2.2	Satisfactory	31.1	32.86

**Figure 2:**
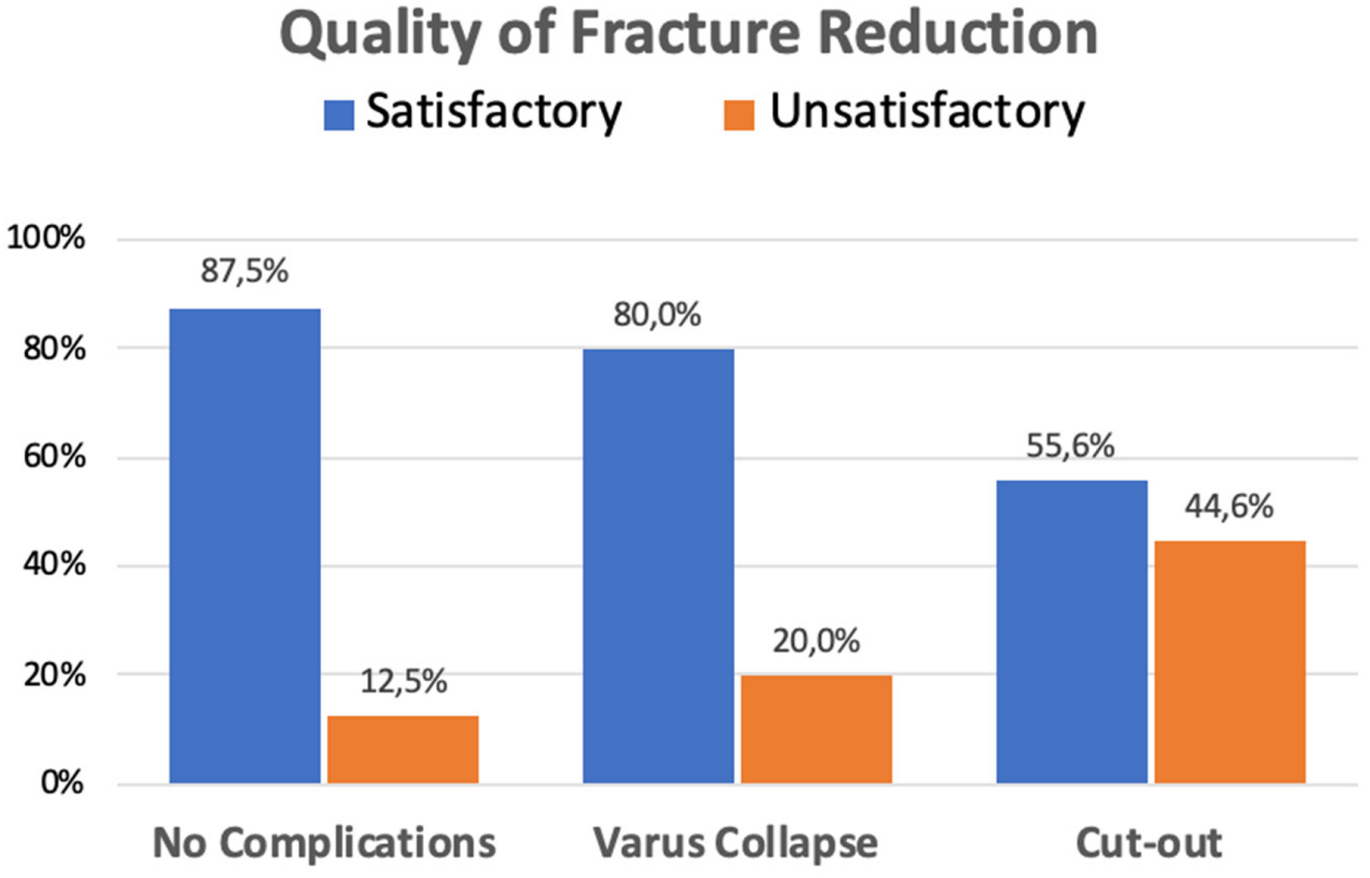
Healing complications according to the quality of fracture reduction.

Table 2 summarizes the surgical outcomes that were identified as associated to varus mobilization and cut-out. The results of the univariate analysis demonstrate that there were statistically significant differences between patients with and without cut-out in the following variables: quality of reduction, length of the lag-screw, fracture collapse according to Doppelt's method, TAD, and CalTAD. In very few patients without healing complications, the quality of the achieved reduction was not satisfactory (12.5%). Those cases with varus collapse had a slightly higher percentage of unsatisfactory reduction (20%). However, patients developing cut-out showed a higher proportion of unsatisfactory fracture reduction compared to patients without healing complications (44%; *p* = 0.022) (Figure 2).

**Table 2:** Descriptive analysis of variables related to surgery.

	Total sample	No complications group (A)	Varus collapse group (B)	Cut-out group (C)	A vs. B *p*-value	A vs. C *p*-value	B vs. C *p*-value
TAD (mm), mean ± SD	21.8 ± 7.3	21.3 ± 7.1	23.7 ± 7.5	32.2 ± 6.2	0.262^a^	<0.001^a^	0.006^a^
Median (ICR)	21.4 (8.8)	20.8 (9)	24.0 (7.5)	32.2 (7.0)			
CalTAD (mm), mean ± SD	23.1 ± 7.3	22.6 ± 7.6	25.1 ± 7.9	34.1 ± 6.2	0.262^a^	<0.001^a^	0.00
Median (ICR)	22.6 (9.3)	22.0 (9.5)	25.4 (8.0)	32.8 (7.4)			
Screw length, *n* (%)							
≤95 mm	173 (43.5%)	142 (39.5%)	23 (76.7%)	8 (88.9%)	0.002^b^	0.008^b^	0.652^b^
>95 mm	225 (56.5%)	217 (60.5%)	7 (23.3%)	1 (11.1%)			
Fracture reduction, *n* (%)							
Satisfactory	343 (87.5%)	314 (87%)	24 (80%)	5 (55.6%)	0.377^b^	0.022^b^	0.197^b^
Non-satisfactory	55 (12.5%)	45 (13%)	6 (20%)	4 (44.4%)			
Doppelt (mm)	2.0 (2.2)	1.8 (2.0)	3.5 (6.9)	2.4 (1.1)			

^a^ Mann–Whitney *U-*test.

^b^ Fisher's exact test.

**Figure 3:**
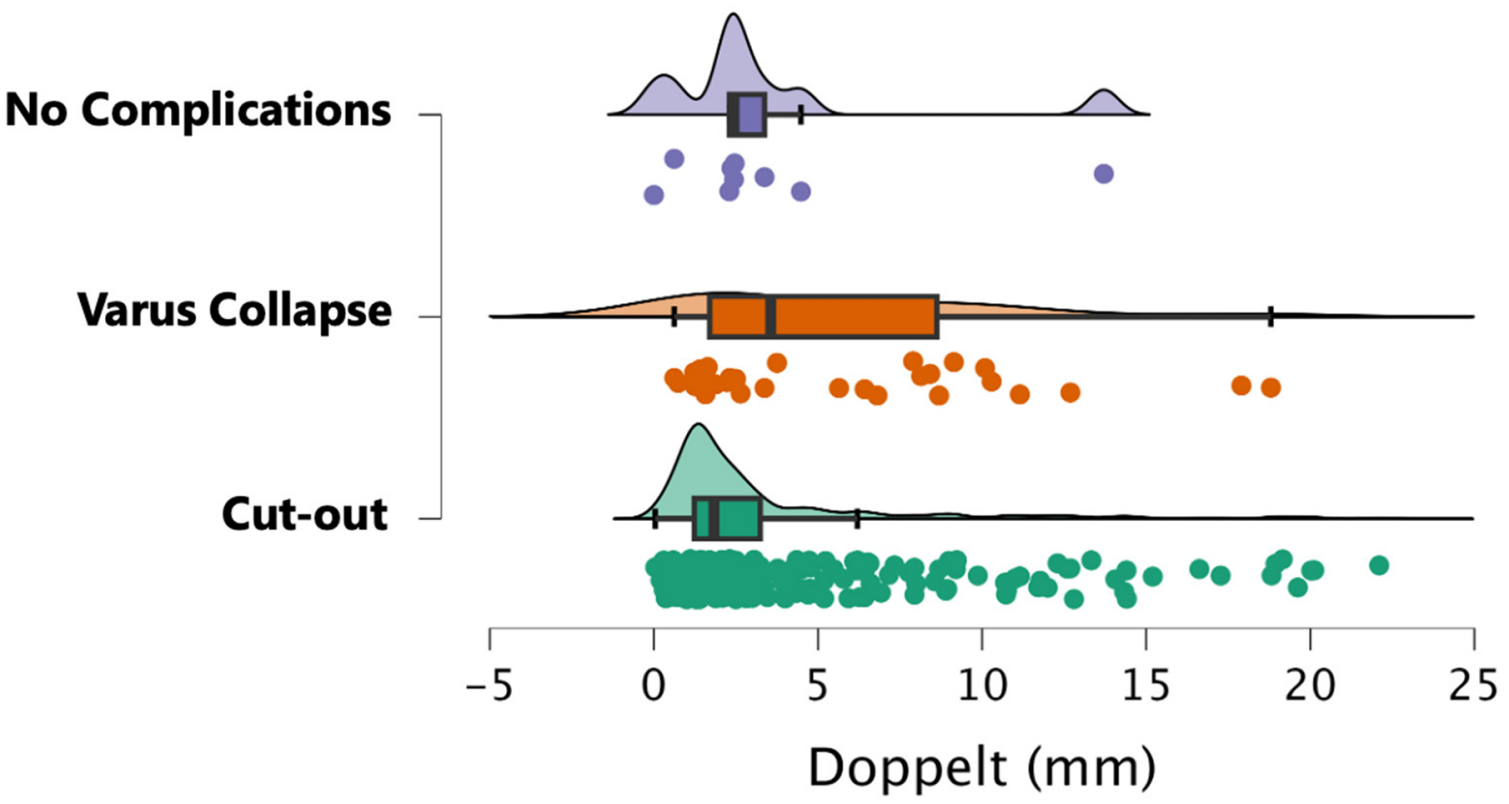
Fracture collapse measured by Doppelt's method according to fracture healing complications.

Concerning fracture collapse assessed by Doppelt's criteria, there were no differences between fractures with no healing complications and those developing cut-out. In both cases, the collapse was minimal [median 1.8 (IQR 2.0) vs. median 2.4 (IQR 1.1) respectively]. As expected, fractures with varus collapse showed a statistically significant greater collapse (median 3.5, IQR 5.9) compared to non-healing complicated cases (*p* = 0.020) (Figure 3).

**Figure 4:**
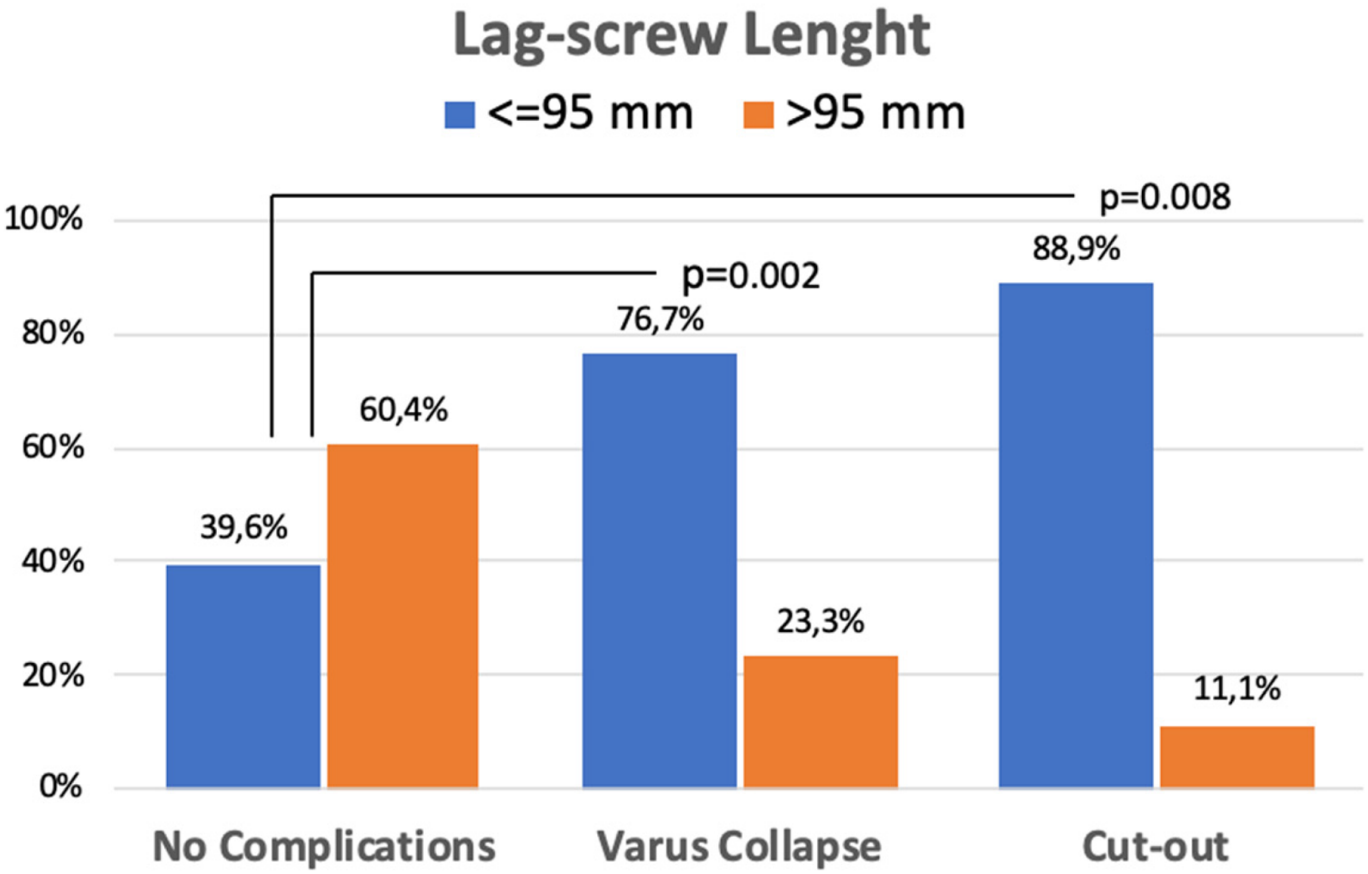
Length of the lag-screw and fracture healing complications.

The AO/OTA classification displayed a statistically significant higher risk of varus collapse in the A3 fracture type (*p *= 0.03). The length of the lag-screw was also found to be related to healing complications. Most cases developing cut-out (88.9%) and those with varus collapse (76.7%) were operated with lag-screws shorter than or equal to 95 mm. In both cases, the differences were statistically significant compared to patients without healing complications (*p* = 0.008 and *p* = 0.002, respectively) (Figure 4).

**Figure 5:**
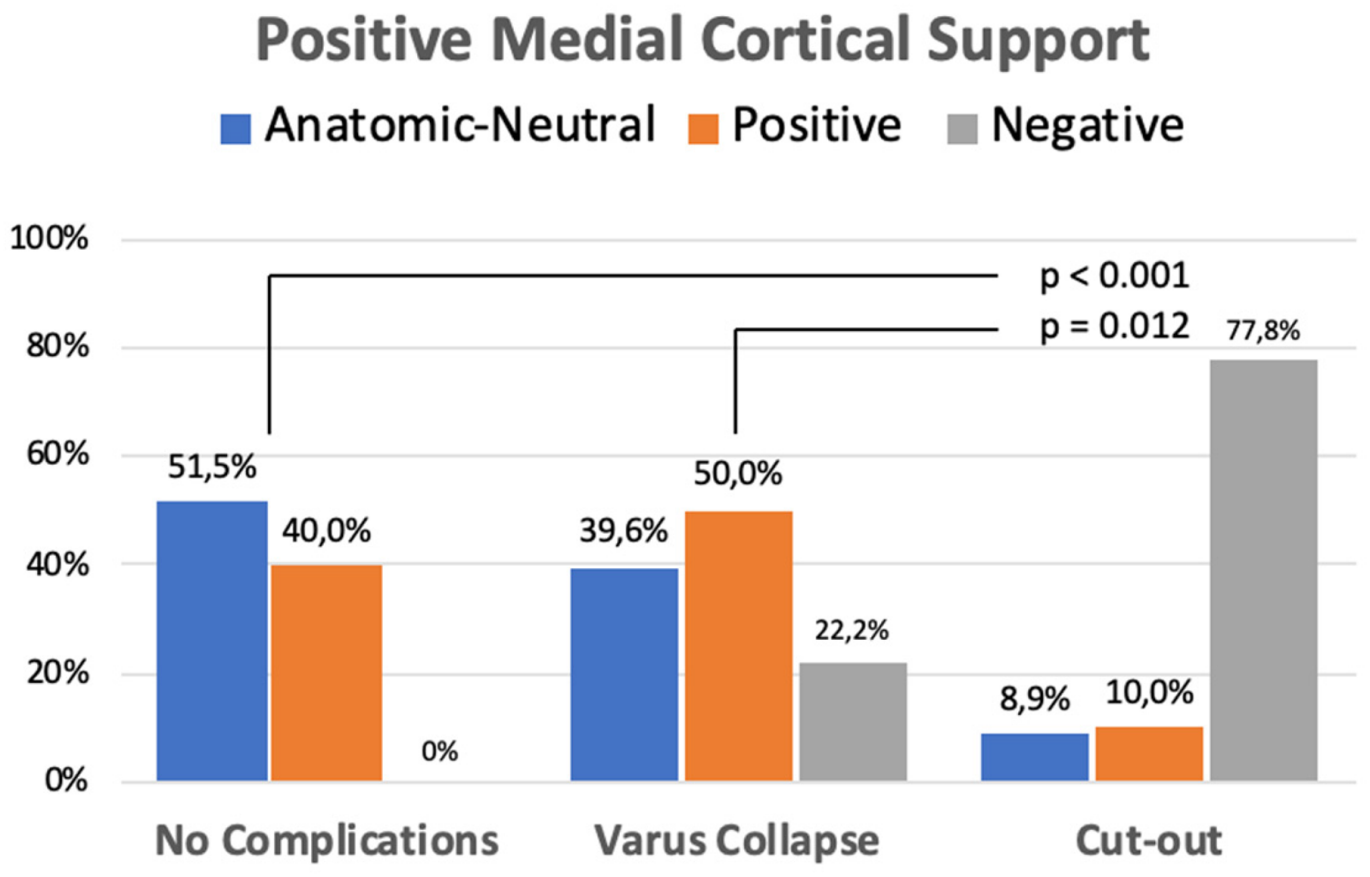
Position of the medial cortical support in the healing complications group.

When the occurrence of mechanical complications was analyzed according to the position of the MCS, some interesting results were observed. There were statistically significant differences between the cut-out group and the non-complicated or varus collapse group in the distribution of postoperative position of the MCS (Figure 5). Varus collapse and cut-out were only found in cases of negative MCS (22.2% and 77.8%, respectively).

**Figure 6:**
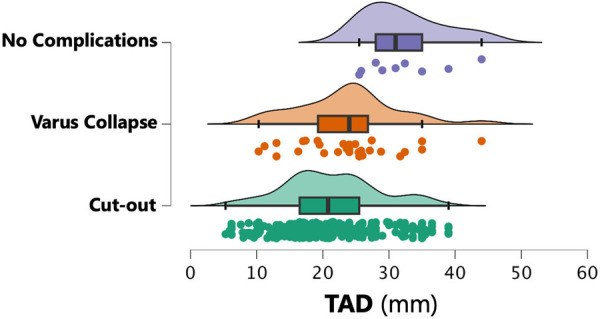
TAD measurements according to the healing complications group.

**Figure 7:**
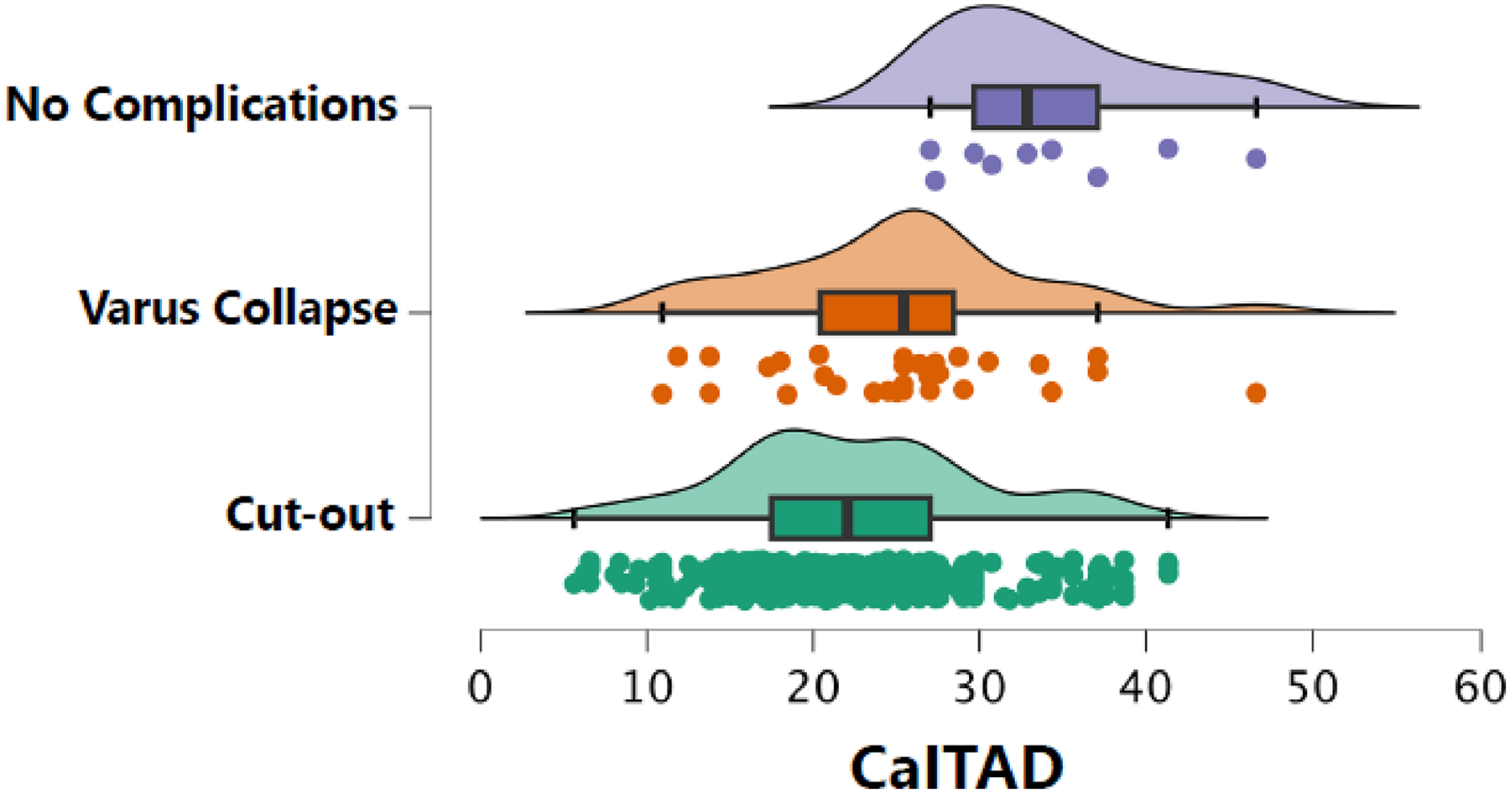
CaITAD measurements according to the healing complications group.

In the no healing complications group, the median TAD was 20.8 mm (IQR 9.0). There were no differences with the TAD values found in patients with varus collapse. However, in the cut-out group, the median TAD was 32.2 (IQR 7.0; *p* < 0.001 compared to the no healing complications group and *p* < 0.01 in comparison to the varus deformity group) (Figure 6). Similarly, while the median CalTAD in the non-healing complications group was 22.0 mm (IQR 9.5), the median CalTAD in the cut-out group was 32.8 mm (IQR 7.4; *p* < 0.001 compared to the non-healing complications group and *p* < 0.01 in comparison to the varus deformity group). There were no statistically significant differences in the mean CalTAD between the two groups without cut-out (Figure 7).

**Figure 8:**
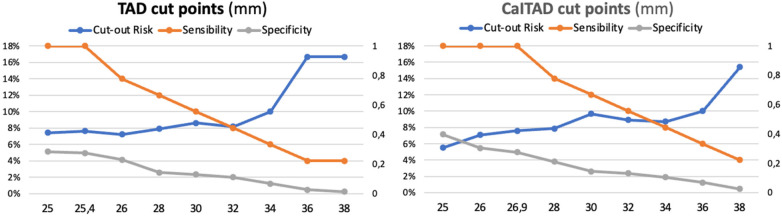
Patients at risk of cut-out: sensibility and specificity of different TAD and CalTAD threshold values.

The application of the Youden test to detect the highest value of sensitivity and specificity showed that the best cut-off values are 25.4 mm for TAD and 26.9 mm for CalTAD. These new cut-offs effectively intercepted all the lag-screw cut-outs encountered in our study. Figure 8 shows the percentage of patients at risk of cut-out, and the sensibility and specificity of different TAD and CalTAD threshold values. Both sensibility and specificity severely decrease as threshold points increase.

The findings from the multivariate analysis revealed that only TAD showed independent significance concerning cut-out. This relationship was established based on both absolute TAD values and a TAD limit of 25 mm, with statistical significance observed in both instances (*p* < 0.001). The multivariate logistic regression model, considering TAD >25 mm and CalTAD of 25 mm, resulted in an odds ratio (OR) of 2.453 [95% confidence interval (CI) 0.864–4.042] for TAD (*p* < 0.001), and an OR of 2.636 (95% CI 1.045–4.227) for CalTAD. Furthermore, when the multivariate logistic regression model considered TAD >25.4 mm and CalTAD >26.9 mm, it yielded an OR of 3.911 (95% CI 1.058–6.763) (*p* < 0.001) for the former and an OR of 3.904 (95% CI 1.051–6.756) (*p* < 0.001) for the latter.

## Discussion

The mechanical failure of osteosynthesis leading to cut-out of the implant is the most feared complication after cephalomedullary nailing of proximal femoral fractures (3, 4, 9, 20). Cut-out has a great impact on functional recovery as it causes restricted mobility, which determines life expectancy in elderly patients (21, 22). Numerous authors have attempted to define cut-out predictors to prevent this complication, but clear evidence is still lacking (23–26). Multiple variables have been evaluated, and many risk factors have been proposed, but the distinction between a fracture that will consolidate and one that will fail is still unclear and requires further studies.

In the current study of 398 cases, age, sex, fracture side, and ASA grade were not related to cut-out, confirming previous studies (16, 22, 27). Fracture patterns most related to cut-out occurrence were unstable, but not those of greater instability according to the AO classification. The literature regarding the relationship between fracture pattern and cut-out occurrence is not conclusive. Kashigar et al. (9) demonstrate that there is no statistically significant relationship of cut-out with the fracture type, while Bojan et al. (20) differ and establish a statistically significant relationship with the A3.3 and B21 fracture patterns. Mavrogenis et al. (3) suggest that the most unstable fracture patterns are a risk for cut-out occurrence because they tend to suboptimal reduction, and screw misplacement into the femoral head.

The malposition of the cephalic screw into the femoral head has been one of the most studied parameters in the literature, being pointed out by many authors as the main cause of implant mechanical failure (17, 21, 22, 27). Nevertheless, the ideal position of the screw in the femoral head has been a matter of controversy in the literature. The central position in lateral projection and the central-inferior position in anteroposterior projection are the most recommended by different authors as they maximize mechanical rigidity and the load necessary for implant failure (1).

Baumgartner's TAD has been globally accepted as the reference measure for screw placement in the femoral head. High TAD values have been a significant predictor of cut-out after cephalomedullary nailing (1, 15, 17, 22, 27). However, some studies suggest that cut-out complications are very infrequent, even in patients with high TAD, if the lag-screw was positioned inferior in the head and neck (4, 5). It has been found that the inferior placement of the lag-screw gives the highest axial and torsional stiffness (17, 27). Most of the previous studies proposed a TAD of <25 mm as a reference to reduce implant mechanical failure (3, 16, 28, 29). Our study is in accordance with these previous findings since there was a statistically significant relationship between TAD values higher than 25 mm and the cut-out in both univariate and multivariate analyses. The best TAD cut-off value with highest value of sensitivity and specificity was 25.4 mm, effectively intercepting all the lag-screw cut-outs encountered in our study.

Nevertheless, there are studies that state that TAD is not the main factor in preventing cut-out since it is not supported by clinical evidence (14, 23, 30). In fact, Yam et al. raised the traditional TAD cut-off from 25 to 27 mm (31), and others have reported that a limit of 25 mm should not be established as a clear predictor of osteosynthesis failure (9, 31). Recently, Caruso et al. (27) suggested that there are reasonable grounds for raising the TAD cut-off from 25 to 34.8 mm. Some authors claim that TAD measurements should be modified for a better prediction of cut-out of lag-screws in trochanteric hip fractures (28, 32).

CalTAD measures were introduced in the literature as the other relevant parameter with a predictive value for cut-out (24, 26). This measurement helps understand that a central-inferior screw position is related to a decrease in cut-out occurrence. However, no definitive CalTAD cut-off has yet been established (15, 18), and the superiority of CalTAD over TAD in predicting cut-out is under controversy. Recent studies found that the value of CalTAD seems to be more effective than TAD in predicting the risk of cut-out after cephalomedullary nailing (9, 17, 27, 30, 33, 34).

In the current research, CalTAD was significantly related to cut-out only in the univariate analysis, but not in the multivariate study. This result is probably because the multivariate statistical analysis tends to filter repetitive information, with CalTAD being partially captured by TAD. However, in a recent series, CalTAD was the only variable with a significant correlation with mechanical failure in a multivariate analysis (33). These contradictory results suggest that perhaps some other additional factors not assessed by CalTAD measurements have an influence on the mechanical failures after cephalomedullary nailing in hip fractures.

The advantage of CalTAD over TAD is that it detects the difference between inferior and superior positioning in the AP view, but it does not evaluate the anterior or posterior position in the lateral plane. Lower CalTAD values indicate that the lag-screw is in the inferior aspect of the femoral head and this position reduces the risk of cut-out (22, 27). In fact, the lower portion of the femoral neck has greater bone density and therefore a strong biomechanical resistance to load. In contrast, other studies consider that the correct screw position is central-central (3, 13, 25).

Bone quality has been another factor that some authors have assessed as a predictor of mechanical failure (3, 18). However, radiographic methods to estimate the degree of osteoporosis, such as the Singh index, do not clearly correlate with true bone density (35). Nonetheless, this was not a parameter evaluated in the current study.

The quality of reduction has been another parameter to consider as it is involved in the occurrence of cut-out, leading to controversies and debates. Kashigar et al. (9) observed a significant relationship in univariate analysis between varus reduction, compared to the contralateral hip, and cut-out. However, these authors did not find a significant relationship between reduction according to the Baumgartner method and cut-out. On the contrary, a higher percentage of cut-out has been found to be associated with poor fracture reductions (27), and a good quality of reduction reduced the risk of mechanical failure of the cephalomedullary implants in both univariate and multivariate analysis (21).

In 2015, Chang et al. described a concept called PMCS to assess the achieved reduction in intertrochanteric fractures treated with intramedullary nailing (12). PMCS is defined as the medial cortical portion of the head–neck fragment that is displaced and placed superomedially relative to the medial cortex of the femoral shaft. The authors describe this reduction as a key element for the stability of reconstruction in unstable fractures. The statistical data are very conclusive in this regard.

The present research demonstrates that an unacceptable reduction according to Baumgartner's method has excellent outcomes regarding the prevention in cut-out occurrence. Perhaps this finding could highlight a certain advantage of intramedullary implants over extramedullary ones, on which Baumgartner bases the TAD, in terms of reduction, as unstable fracture cases present certain mechanical advantage. Undoubtedly, more studies are needed in this regard to appreciate this assessment of reduction quality.

The current study has some limitations, mainly related to its retrospective design and the small number of cut-outs found in a relatively large series of trochanteric hip fractures. The value of findings arises from the particularity that all patients were operated on by the same single surgeon. Furthermore, body mass and bone mineral density are variables that were not documented and undoubtedly could improve the quality of the study.

In summary, the current study clearly defines a statistical relationship between TAD and the occurrence of cut-out. The relationship between CalTAD and cut-out occurrence was less significant than TAD in multivariate studies. The type of fracture was not a clear predictor of cut-out occurrence. Reduction quality was not directly related to cut-out since in 55% of cut-out cases the reduction was considered good according to Baumgartner's method. The results of this study suggest that cut-out can be prevented with adequate preoperative planning, comprehensive fracture evaluation, and precise surgical technique.

## Data Availability

The raw data supporting the conclusions of this article will be made available by the authors upon request, without undue reservation.
